# Adsorption of Cd^2+^ by *Lactobacillus plantarum* Immobilized on Distiller’s Grains Biochar: Mechanism and Action

**DOI:** 10.3390/microorganisms12071406

**Published:** 2024-07-11

**Authors:** Guangxu Zhu, Xingfeng Wang, Ronghui Du, Shuangxi Wen, Lifen Du, Qiang Tu

**Affiliations:** 1 College of Biology and Environment Engineering, Guiyang University, Guiyang 550005, China; 2 State Key Laboratory of Microbial Technology, Shandong University, Qingdao 266237, China

**Keywords:** biochar, *Lactobacillus plantarum*, immobilized microbial technology, cadmium (II), adsorption mechanism

## Abstract

Immobilized microbial technology has recently emerged as a prominent research focus for the remediation of heavy metal pollution because of its superior treatment efficiency, ease of operation, environmental friendliness, and cost-effectiveness. This study investigated the adsorption characteristics and mechanisms of Cd^2+^ solutions by *Lactobacillus plantarum* adsorbed immobilized on distiller’s grains biochar (XIM) and *Lactobacillus plantarum*–encapsulated immobilized on distiller’s grains biochar (BIM). The findings reveal that the maximum adsorption capacity and efficiency were achieved at a pH solution of 6.0. Specifically, at an adsorption equilibrium concentration of cadmium at 60 mg/L, XIM and BIM had adsorption capacities of 8.40 ± 0.30 mg/g and 12.23 ± 0.05 mg/g, respectively. BIM demonstrated noticeably greater adsorption capacities than XIM at various cadmium solution concentrations. A combination of isothermal adsorption modeling, kinetic modeling, scanning electron microscopy–energy dispersive X-ray spectroscopy, X-ray diffractometer (XRD), and Fourier-transform infrared spectroscopy (FTIR) analyses showed that cadmium adsorption by XIM primarily involved physical adsorption and pore retention. In contrast, the adsorption mechanism of BIM was mainly attributed to the formation of Cd(CN)_2_ crystals.

## 1. Introduction

Cadmium (Cd) is an extremely toxic and non-essential heavy metal element for humans. It has been distinguished as a class I human carcinogen [1] and is listed by the United Nations Environment Programme (UNEP) as the top hazardous chemical among the 12 globally significant substances [2]. Cd is known for its elevated persistence, non-degradability, and mobility in the natural environment [3]. Discharging cadmium-containing industrial wastewater from electroplating, mining, and smelting industries into water bodies has substantially increased Cd pollution, directly endangering human and wildlife health [4,5,6]. Consequently, tackling Cd contamination in water environments has become a critical and enduring concern in environmental research.

Among the remediation technologies for heavy metal pollution, the microbial method is an environmentally friendly, efficient, and economical approach to eliminating heavy metals using biochemical, bioprecipitation, and biomineralization pathways [7,8]. Previous studies have demonstrated the efficacy of *Lactobacillus plantarum* in removing heavy metal ions such as cadmium and lead. Zhai et al. (2014) used in vitro screening to identify a strain of *Lactobacillus plantarum* with excellent cadmium adsorption capabilities. They employed mice as an animal model and conducted acute and chronic cadmium exposure experiments. Their findings showed reduced cadmium content in tissues and significant mitigation of cadmium toxicity and oxidative damage [9]. Ameen et al. (2020) reported a strain of *Lactobacillus plantarum* MF042018 that exhibited a high degree of resistance, up to 500 and 100 ppm for nickel and chromium, respectively, thus demonstrating a high absorption capacity for Cd^2+^ and Pb^2+^. Despite the promising outcomes of using microorganisms for heavy metal adsorption in wastewater, challenges arise due to the small size and low density of microbial particles, making solid-liquid separation challenging and hindering the removal of suspended organisms [10,11,12]. These obstacles restrict the widespread applications of biosorption technology.

In recent years, the treatment of heavy metal-contaminated wastewater with immobilized microbial technology has garnered extensive attention because of its notable advantages of high efficiency and stability [13]. This innovative approach includes confining free and dispersed microorganisms within a limited space using physical or chemical methods to enhance the concentration of microbial cells. This, in turn, sustains high biological activity, making them conducive to subsequent utilization [14]. Immobilized microbial technology offers significant advantages over free microbial technology. Immobilized carriers serve as buffers, enabling microorganisms to adapt more effectively to extreme pH levels and toxic environments [15,16]. Selecting suitable carriers is crucial in immobilization technology since these carriers retain bacteria and exhibit excellent permeability, allowing the easy penetration of heavy metal ions. As such, the choice of carriers plays a pivotal role in the success of immobilization technology.

In general, ideal microbial immobilization carriers are characterized by high porosity, good stability, non-toxicity, and affordability [14]. Biochar, derived from the thermal decomposition of biomass under anoxic or anaerobic conditions, possesses a large specific surface area and porous structure [17]. These features help reduce cell dispersion and offer ample space for microbial survival. The presence of elements, such as carbon, oxygen, potassium, nitrogen, and phosphorus, in biochar provides essential nutrients for microorganisms [18,19]. Moreover, the abundant functional groups on the biochar surface, including carboxyl and carbonyl groups, exhibit a strong affinity to microbial cell membranes, facilitating effective biochar loading with microorganisms [20]. Therefore, biochar stands out as an excellent carrier. With its large specific surface area, porous structure, high pH, and active functional groups, biochar proves highly effective in adsorbing several heavy metal pollutants in water, making it a popular choice for managing wastewater contaminated with cadmium and other heavy metals [21,22,23]. However, there is a lack of research on the adsorption of heavy metal pollutants by immobilized *Lactobacillus plantarum* using biochar. This paucity of studies presents an intriguing avenue for future studies in the field.

Guizhou is an important base for Maotai-flavor liquor production in China. In recent years, the scale of the Maotai-flavor liquor industry in Guizhou Province has been expanding and developing rapidly. The production of distiller’s grains, a by-product of organic residue produced in the process of liquor-making, has also increased year by year. According to statistics, the annual output of distiller’s grains in Guizhou Province is about 3000,000 tons [24]. However, the recycling and utilization of distiller’s grains has not received much attention. Apart from a small portion being used as raw materials for feed and organic fertilizers, it is generally treated as waste for landfilling, dumping, or incineration, leading to resource wastage and potential environmental pollution. Since distiller’s grains contain a large amount of protein, vitamins, and cellulose and have a high carbon content, they can be efficiently converted into biochar.

According to the methods of binding between microorganisms and carrier biochar, immobilization methods can be divided into the adsorption method, encapsulation method, cross-linking method, covalent binding method, etc. [25]. Adsorption immobilization relies on physical adsorption interactions such as hydrogen bonds and charge forces between microorganisms and the surface of carrier biochar to bind together. Typically, the immobilization involves mixing sterilized carrier materials and microbial solution in a proportion for a certain period, followed by centrifugal separation to obtain immobilized microorganisms. This method is simple and convenient to operate, allows for easy exchange of microbial substances, and is environmentally friendly and cost-effective, making it a commonly used method in bioremediation processes [26]. Encapsulation immobilization uses a fixative to encapsulate microorganisms in capsules, microspheres, or membrane forms to achieve immobilization. Currently, polyvinyl alcohol and sodium alginate are commonly used fixatives for encapsulation. This method stabilizes the microbes inside the carrier, reducing leakage, and protects them from external interference, thus preserving their vitality well. It is simple, low-cost, and suitable for large-scale production [27]. Cross-linking immobilization utilizes chemical agents that can react with surface groups on microorganisms to cross-link them into a network structure, thereby immobilizing the microorganisms, similar to a flocculation reaction. This method has strong binding effects but is complex to operate, demanding reaction conditions and stringent requirements for the carrier, with the risk of microbial inactivation and higher costs [28]. Covalent binding immobilization involves activating functional groups on the immobilized carrier’s surface to form covalent bonds with microorganisms, enabling a strong association between the carrier and microorganisms. This method allows for close linkage between microorganisms, providing high stability, but it involves complex steps, challenging control of reaction conditions. Therefore, the most commonly used methods for immobilizing microorganisms currently are adsorption immobilization and encapsulation immobilization [29].

To reveal the adsorption characteristics of Cd^2+^ by *Lactobacillus plantarum* immobilized on biochar, this study used biochar prepared from distiller’s grains under high-temperature and limited oxygen conditions as the carrier. *Lactobacillus plantarum* isolated from garbage enzyme was utilized as the inoculated bacterial community. Two types of immobilized bacterial biochar were prepared via adsorption immobilization and encapsulated immobilization methods, respectively, to compare their adsorption characteristics on Cd^2+^. The research delved into the impacts of the dosed amount of absorbent material, the solution’s pH, initial Cd^2+^ concentration, and adsorption duration on the adsorption efficiency. Various analyses such as adsorption kinetics, adsorption isotherms, X-ray diffractometer (XRD), and Fourier-transform infrared (FTIR) analysis were conducted to understand the adsorption process and mechanism of Cd^2+^ by *Lactobacillus plantarum* immobilized on biochar. These analyses aimed to yield fundamental data and theoretical insights into treating Cd-contaminated wastewater using *Lactobacillus plantarum* immobilized on biochar.

## 2. Materials and Methods

### 2.1. Biochar Preparation

**Figure 1:**
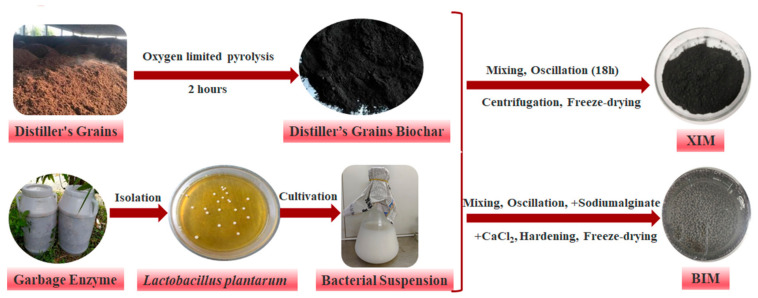
Flow charts of ordinary distiller’s grains biochar, XIM, and BIM preparation.

The raw materials of rice husk distiller’s grains were taken from a Maotai-flavor liquor factory in Renhuai City, Guizhou Province. The raw materials of the distiller’s grains were crushed, sieved, compacted, and packed into aluminum boxes, sealed with tin foil, and heated in a muffle furnace at a heating rate of 10 °C/min. The temperature was raised to 450 °C and maintained for 2 h. After natural cooling, the material was extracted and ground through a 100-mesh sieve to obtain ordinary distiller’s grains biochar (BC, Figure 1).

The tested strains were *Lactobacillus plantarum* with Cd tolerance preserved in the laboratory. *Lactobacillus plantarum* belongs to the Lactobacillus family. The bacterial species are straight or curved rod-shaped, with an optimal growth temperature of 30–37 °C. It is a Gram-positive facultative anaerobic bacterium that grows at a pH value of 4.5–9.5, with an optimal pH value of around 6.5. This strain was screened and extracted from garbage enzyme prepared by fermentation of fruit and vegetable waste. Our previous study found that this strain had high tolerance and adsorption to lead and cadmium and could reduce the concentration of Pb^2+^ and Cd^2+^ in aqueous solutions. The preserved *Lactobacillus plantarum* was inoculated into the sterilized 100-mL Mann–Rogosa–Sharpe (MRS) Broth liquid medium at an inoculum rate of 1%. The mixture was then kept in an incubator set at 35 °C for an 18-h expansion culture. The amplified organisms were inoculated into the prepared 1 L MRS liquid medium at an inoculation rate of 1%. Following incubation, the culture was centrifuged at 3500 r/min for 5 min at 4 °C, washed, and centrifuged twice with 0.85% sterile saline. Subsequently, the organisms were diluted with 0.85% sterile saline until reaching an optical density (OD) value of 1.0, and a bacterial suspension was then prepared for future use.

After weighing 20 g of sterilized distiller’s grains biochar, the biochar was mixed with the bacterial suspension in 500-mL conical flasks at a ratio of 1:10 (*m*:*V*) units. The mixture was then subjected to a shaking culture at 30 °C and 180 r/min for 18 h. Following this, the culture underwent centrifugation at 3500 r/min for 5 min. The lower layer of precipitation was washed twice with 0.85% sterile saline, followed by centrifugation. Afterward, the material underwent vacuum freeze-drying, resulting in the solid being utilized for the *Lactobacillus plantarum* adsorbed immobilized on the distiller’s grains biochar (XIM, Figure 1).

After weighing 20 g of sterilized distiller’s grains biochar, it was mixed with the bacterial suspension at a ratio of 1:10 (*m*:*V*) in a 500-mL conical flask. The mixture was then kept in a shaker set at 30 °C and 180 r/min for 18 h. Next, the contents were combined with a 4% (*m*:*V*) sodium alginate [(C_6_H_7_NaO_6_)_n_] solution in equal volume. Following thorough mixing, the mixture was slowly added drop by drop into a sterilized 400 mL 4% calcium chloride (CaCl_2_) solution using a syringe. Stirring it drop by drop allowed the formation of immobilized small particles that were subsequently left in the CaCl_2_ solution to harden for 12 h. The particles were then washed thoroughly with sterile water before undergoing vacuum freeze-drying. This process resulted in forming *Lactobacillus plantarum* encapsulated and immobilized on the distiller’s grain biochar (BIM, Figure 1).

### 2.2. Adsorption Experiment Design

Cadmium chloride (CdCl_2_) and ultrapure water were applied to prepare a mother liquor containing a Cd^2+^ concentration of 5000 mg/L. Subsequently, sodium nitrate (NaNO_3_) was employed as the background electrolyte with a Na⁺ ionic strength of 0.01 mol/L. The mother liquor was diluted to obtain Cd^2+^ solutions with concentrations of 5, 10, 20, 40, 60, and 80 mg/L. The pH of these solutions was adjusted using 0.1 mol/L of sodium hydroxide (NaOH) solution and 0.1 mol/L of nitric acid (HNO_3_) solution to achieve the desired pH levels.

A specified amount of XIM and BIM was introduced into a 50-mL centrifuge tube, followed by 20 mL of Cd^2+^ solution with varying concentrations. The tube was then sealed and positioned on an intelligent thermostatic oscillator. After being shaken for different durations at 25 °C, the contents were centrifugated at 4000 rpm for 5 min and filtered using a filter tip of 0.22 μm. To investigate the adsorption capacities of XIM and BIM on Cd^2+^ in solution, parameters such as the dosage of XIM and BIM, solution pH, initial Cd^2+^ concentration, and reaction time, among others, were varied (Table 1). Experiments were conducted under different conditions, with four parallel groups and a control group. The concentrations of Cd^2+^ were determined using inductively coupled plasma optical emission spectrometry (ICP-OES, Optima 5300DV, PerkinElmer, Waltham, MA, USA) and inductively coupled plasma mass spectrometry (ICP-MS, ELAN DRC-e, PerkinElmer, Waltham, MA, USA).

The reagents used in this study include (C_6_H_7_NaO_6_)_n_, CaCl_2_, MRS broth medium, NaCl, CdCl_2_, NaNO_3_, NaOH, and HNO_3_. Except for HNO_3_, which is of superior purity, all other reagents are analytical grade. Water used for dilution and dissolution was purified using a Millipore deionizing system at 18.2 MΩ.

### 2.3. Characterization of Biochar

A scanning electron microscope (SEM, ZEISS GeminiSEM 300, Oberkochen, Germany) was used to capture morphological changes of XIM and BIM at different magnifications. An energy-dispersive X-ray spectroscopy (EDS, OXFORD Xplore, Oxford, UK) mapping spectrometer was employed to conduct elemental composition analysis on their surfaces. The X-ray powder diffractometer (XRD, Rigaku SmartLab SE, Tokyo, Japan) was utilized to determine the crystalline substances in XIM and BIM; the crystal characteristic peaks of XIM and BIM were compared with standard cards in Jade6.0 to identify the solid-phase crystal components. A Fourier-transform infrared (FTIR, Thermo Scientific Nicolet iS20, Cupertino, CA, USA) spectrometer was employed to analyze the functional groups on the surfaces of XIM and BIM and to create FTIR spectra for examining changes in surface functional groups. By using an automatic specific surface area and porosity analyzer (APSP 2460, Irving, TX, USA), nitrogen adsorption and desorption tests were conducted on the sample under 77.3 k liquid nitrogen conditions. After the instrument analysis was completed, the isothermal adsorption and desorption curve was obtained. The total specific surface area of the biochars was obtained by the Barrett Emmett Telle (BET) method, and the pore volume and pore size of the material were obtained by the Barrett Joyner Halanda (BJH) method.

### 2.4. Data Analysis

#### 2.4.1. Calculation of Adsorption Capacity and Adsorption Efficiency

The adsorption amount (*q_e_*) and adsorption efficiency (η) of immobilized *Lactobacillus plantarum* wine lees biochar on Cd^2+^ were calculated as follows [30]:

(1) $$\begin{array}{l} q_{e}=\frac{V(C_{0}\;-\;C_{t})}{m} \end{array}$$

(2) $$\begin{array}{l} \eta =\frac{C_{0}{\;-\;C}_{t}}{C_{0}}\times 100\% \end{array}$$

where, *q_e_* is the amount of cadmium adsorbed per unit of biomass at equilibrium, mg/g; *V* is the solution volume, L; *C*_0_ and *C_t_* are the mass concentration of Cd^2+^ in the solution at the initial moment (before adsorption) and at moment *t* (after adsorption), respectively, mg/L; and *m* is the mass of adsorbent, g. The adsorbent was used for the adsorption of Cd at the initial moment (before adsorption) and at the moment t (after adsorption), respectively.

#### 2.4.2. Isothermal Adsorption Model

Langmuir and Freundlich model equations were used to fit the data. The adsorption performance of *Lactobacillus plantarum* immobilized on distiller’s grains biochar was analyzed with the following equation [31]:

Langmuir equation:

(3) $$q_{e}=\frac{q_{m}K_{L}C_{e}}{1+K_{L}C_{e}}$$

Freundlich equation:

(4) $$q_{e}=K_{F}C_{e}^{\frac{1}{n}}$$

where: *q_e_* is the average adsorption capacity of *Lactobacillus plantarum* immobilized on distiller’s grains, mg/g; *q_m_* is the maximum adsorption capacity, mg/g; *K_L_* is the Langmuir model constant; *C_e_* is the mass concentration of Cd^2+^ at adsorption equilibrium, mg/L; *K_F_* is the Freundlich model constant; and n is the dimensionless constant.

#### 2.4.3. Kinetic Model

Pseudo-first-order kinetics Equation (5), pseudo-second-order kinetic equation Equation (6), and Elovich model Equation (7) were used to fit the data of Cd^2+^ adsorption by XIM and BIM to analyze the adsorption performance of *Lactobacillus plantarum* immobilized on distiller’s grains as shown in the following equations [32]:

(5) $$q_{t}=q_{e}(1-e^{-K_{1t}})$$

(6) $$q_{t}=\frac{K_{2}q_{e}^{2}t}{1+k_{2}q_{e}t}$$

(7) $$q_{t}=\frac{1}{\beta }\ln(\alpha \beta t+1)$$

where *q_e_* is the average adsorption amount of *Lactobacillus plantarum* immobilized on distiller’s grains, mg/g; *q_t_* is the adsorption amount at the moment *t*, mg/g; *K*_1_ is the rate constant of pseudo-first-order kinetics, min^−1^; *K*_2_ is the rate constant of pseudo-second-order kinetics, g/(mg-min); and *α* and *β* are the rate of adsorption (mg/g) and desorption constant (g/mg).

## 3. Results

### 3.1. Analysis of Physical and Chemical Properties of Biochar

The basic characteristics of regular distiller’s grains biochar and two types of immobilized *Lactobacillus plantarum* distiller’s grains biochars are presented in Table 2. The pH values of XIM and BIM were recorded as 7.23 and 7.78, respectively, which are lower than that of BC (9.94). During the pyrolysis process, the continuous decomposition and release of biological acids from the distiller’s grains, coupled with the presence of inorganic and alkaline substances in the ash content, contribute to the alkalinity of the distiller’s grains biochar [33]. With the introduction of *Lactobacillus plantarum* onto the biochar surface through multiple washing, along with the production of organic acids by the microorganisms themselves, there is a consequent decrease in the pH levels of the immobilized *Lactobacillus plantarum* biochar.

The specific surface areas of XIM and BIM are both greater than that of BC, with BIM having the highest specific surface area, while the total pore volume and average pore diameter are smaller in XIM and BIM compared to BC. This could be attributed to the *Lactobacillus plantarum* occupying the surface and pores of the biochar, thereby increasing the surface area and taking up space on the biochar surface and within the pores. Consequently, this increase in the specific surface area enhances the adsorption capacity for microorganisms and organic pollutants, reduces porosity, and aids in the retention of pollutants post adsorption.

### 3.2. Effect of Dosage on the Adsorption Performance of Cd^2+^

**Figure 2:**
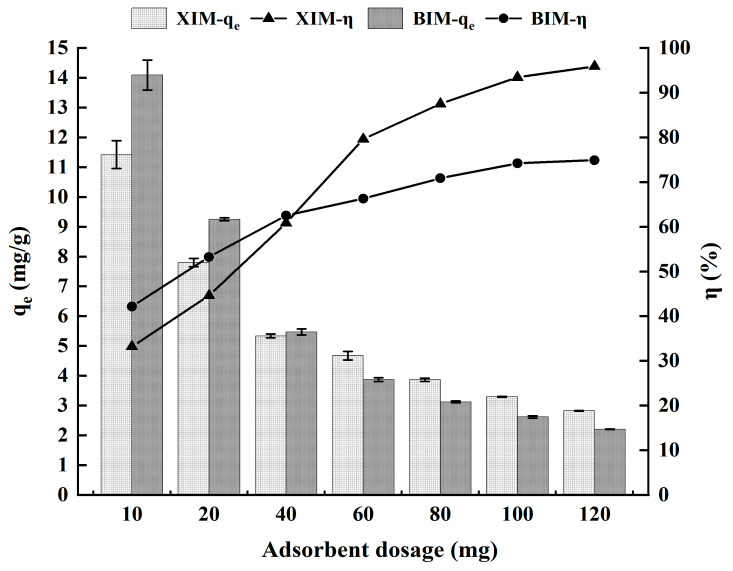
Effect of adsorbent dosage on adsorption characteristics of Cd^2+^.

Figure 2 and Table 3 illustrate the impact of adsorbent material dosage on the experiment. The adsorption behavior of XIM and BIM on Cd^2+^ displays a similar pattern: as the dosage increases, the unit adsorption capacity gradually decreases. In contrast, the adsorption efficiency exhibits an upward trend. The graph shows that at an additional amount of 0.01 g, the unit adsorption capacity of XIM and BIM for Cd^2+^ was at its peak, albeit with lower adsorption efficiency. The adsorption efficiency of both adsorbent materials also increased with further increments in dosage. At a dosage of 0.12 g, the Cd adsorption rate reached 77.91% for BIM and 95.91% for XIM.

Before reaching a dosage of 0.04 g, the adsorption capacity and efficiency of BIM for Cd^2+^ were superior to those of XIM. Subsequently, after the dosage exceeded 0.04 g, the adsorption capabilities and efficiencies of BIM for Cd^2+^ became inferior to those of XIM. The adsorption efficiencies of XIM and BIM on Cd^2+^ were inadequate for incredibly low and overly high dosages; therefore, they did not considerably enhance the adsorption effect. Instead, they escalated costs, and an optimal dosage of 0.05 g was selected for this experiment. Accordingly, 0.05 g was deemed the most effective quantity for subsequent study. At this specific dosage, the adsorption capacity of BIM for Cd^2+^ ranged from 3.87 to 5.47 mg/g, with an adsorption rate of 62.50 to 66.31%. Similarly, the adsorption capacity of XIM for Cd^2+^ ranged from 4.67 to 5.34 mg/g, with an adsorption rate varying from 60.84% to 79.61%. Under the same adsorption test conditions as the present study, Jiang et al. (2021) found that adding 0.05 g of ordinary distiller’s grains biochar resulted in an adsorption capacity of 1.46 mg/g for a 20-mg/L Cd^2+^ solution [34], which was much lower than the adsorption capacity of XIM and BIM for Cd^2+^ in this study.

### 3.3. Effect of pH on the Adsorption Properties of Cd^2+^

**Figure 3:**
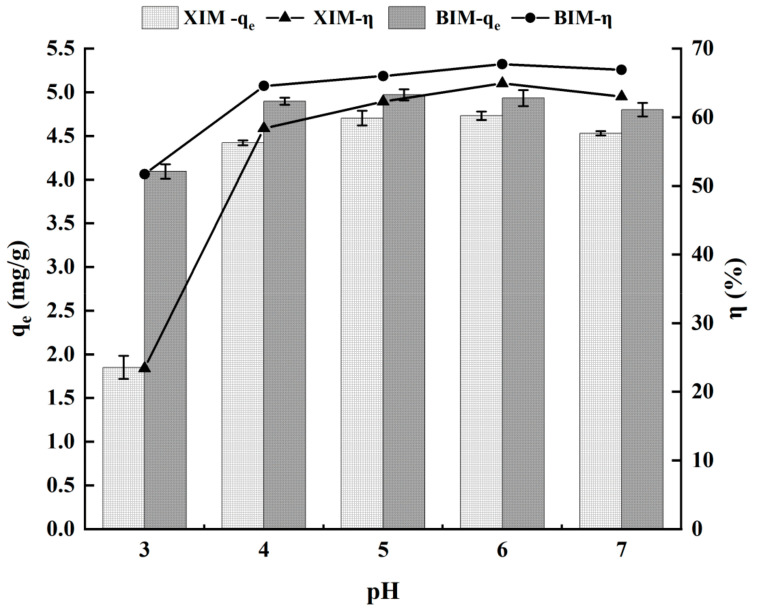
Effect of solution pH on adsorption of Cd^2+^ by adsorbent.

Figure 3 and Table 3 depict the adsorption capacity and efficiency of Cd^2+^ under XIM and BIM under varying pH conditions. At a pH of 3.0, both XIM and BIM exhibited lower adsorption capacity and efficiency. However, equilibrium was reached at pH values of 5.0 and 6.0. In particular, at a pH of 5.0, the adsorption amount was 4.71 mg/g for XIM (with an adsorption rate of 62.29%) and 4.97 mg/g for BIM (with an adsorption rate of 66.00%). At a pH of 6.0, the adsorption amount was 4.73 mg/g for XIM (with an adsorption rate of 64.94%) and 4.93 mg/g for BIM (with an adsorption rate of 67.73%).

Under acidic conditions (low pH), there is competition between a high concentration of H⁺ and Cd^2+^ for intensified adsorption sites. This led to the surface functional groups of XIM and BIM being readily protonated, thereby suppressing the activity of *Lactobacillus plantarum* attached to the biochar surface and inhibiting material adsorption on Cd^2+^. Conversely, with an increase in the solution’s pH, the surface functional groups of XIM and BIM gradually deprotonated, enhancing the negative charge on the surfaces. This alteration facilitated the complexation of surface functional groups and Cd^2+^ in the solution, thereby facilitating Cd^2+^ adsorption by biochar and *Lactobacillus plantarum* [35]. At a pH of 7.0, a notable quantity of OH⁻ in the solution precipitated with Cd^2+^, thus reducing the mobility of heavy metal ions and causing a lower adsorption capacity [36].

### 3.4. Effect of Adsorption Time on the Adsorption Properties of Cd^2+^

**Figure 4:**
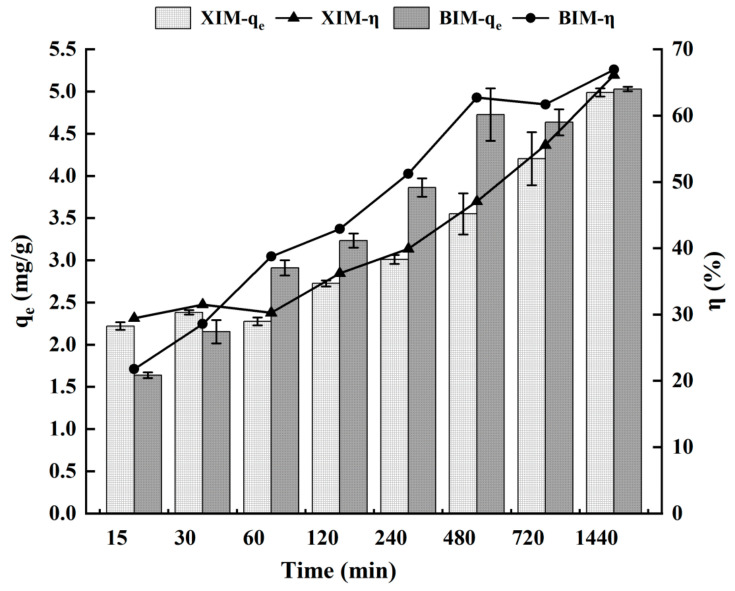
Effect of adsorption time on adsorption of Cd^2+^ by adsorbent.

Figure 4 and Table 3 depict the impact of XIM and BIM on the adsorption of Cd^2+^ over varying durations, ranging from 15 min to 1440 min (24 h). The data indicate that the adsorption of Cd^2+^ by BIM increased over time, with relative equilibrium reaching approximately 8 h into the adsorption reaction, which was similar to ordinary distiller’s grains biochar [34]. At 8 h of adsorption, the adsorption capacities of XIM and BIM reached 71% and 94% of their respective saturation adsorption capacities. This trend might be attributed to the larger specific surface area of BIM, which has a smaller pore diameter and capacity. During Cd^2+^ adsorption, the Cd^2⁺^ ions in the solution predominantly remained on the surface of the spherical biochar BIM due to its greater specific surface area. This led to the surface pores of the biochar being occupied by Cd^2+^ [37]. In addition, the positive charge on the biochar surface increased post-complexation and ion exchange reactions, impeding the further diffusion of Cd^2+^ into the inner spaces of the spheres, ultimately resulting in the adsorption amount of BIM reaching equilibrium [38].

Conversely, the adsorption amount of XIM on Cd^2+^ gradually increased from 15 to 1440 min (24 h), matching that of BIM at 24 h. This observation could be attributed to the larger pore volume and the smaller specific surface area of XIM. Consequently, during the adsorption process, Cd^2+^ first diffused into the biochar surface and then slowly diffused into the pores, slowing down the overall reaction kinetics.

### 3.5. Effect of Cd^2+^ Concentration on Adsorption Performance

**Figure 5:**
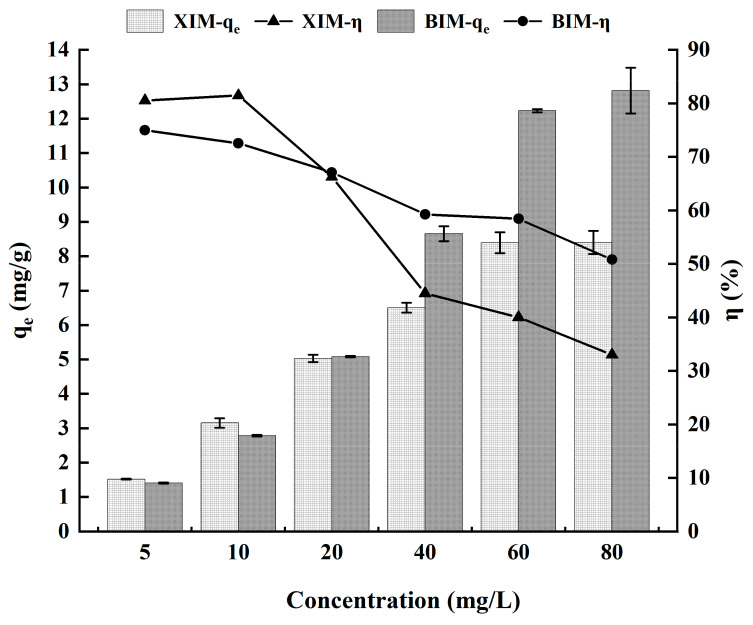
Effect of solution concentration on adsorption of Cd^2+^ by adsorbent.

Figure 5 and Table 3 show the impact of the initial Cd^2+^ solution concentration on the adsorption of Cd^2+^ by XIM and BIM. It also demonstrates that the adsorption amounts of both XIM and BIM tended to stabilize when the Cd^2+^ concentration reached 60 mg/L. At this concentration, the adsorption amounts were 8.39 mg/g for XIM and 12.23 mg/g for BIM. Subsequently, at a concentration of 80 mg/L, the adsorption amounts were 8.40 mg/g for XIM and 12.82 mg/g for BIM. The adsorption amounts for XIM and BIM increased with higher solution concentrations before eventually reaching a plateau.

Furthermore, the adsorption rate decreased with the increase in the solution concentration. This behavior indicates that the rise in initial Cd^2+^ concentration in the solution leads to the gradual saturation of the available adsorption sites on the biochar materials, decreasing the adsorption rate. Overall, both XIM and BIM exhibited effective adsorption of Cd^2+^ across different initial solution concentrations, with adsorption amounts stabilizing with the increase in concentration.

The adsorption amounts of both XIM and BIM increased with the rise in solution concentration and eventually reached a stabilization point. In contrast, the adsorption rates decreased with the increase in the solution concentration. This behavior can be attributed to the fact that at lower solution concentrations, the functional group content and pore size structure of the biochar surface could offer more adsorption sites for cadmium adsorption. Consequently, the adsorption amounts of XIM and BIM for Cd^2+^ increased rapidly with rising solution concentrations. However, as the concentration became too high, the available adsorption sites on the biochar surface became gradually occupied, leading to the saturation of Cd^2+^ adsorption on the biochar. As a result, the immobilization of biochar hindered the activity of *Lactobacillus plantarum*. Consequently, the adsorption no longer significantly increased once the concentration exceeded a certain threshold. The inhibition of *Lactobacillus plantarum* activity due to the immobilization of biochar caused a decrease in the adsorption efficiency when the Cd^2+^ concentration was excessively high. This observation further illustrates that although the initial solution concentration plays a crucial role in adsorption performance, there is an optimal concentration range where the biochar materials can effectively adsorb Cd^2+^ without saturating or inhibiting biological activity [39,40].

### 3.6. Adsorption Isotherm Analysis

**Figure 6:**
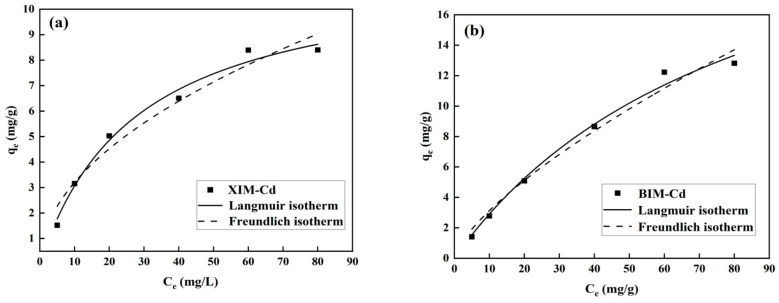
Isothermal adsorption fitting curves of adsorption of Cd^2+^ by XIM (**a**) and BIM (**b**).

Figure 6 illustrates the fitting analysis of the adsorption data using the Langmuir and Freundlich isothermal adsorption models. Figure 6a,b present the impact of initial Cd^2+^ concentration on the adsorption of Cd^2+^ by XIM and BIM. As the Cd^2+^ concentration increases, the adsorption amount of both XIM and BIM also increases. However, the rate of increase gradually diminishes, which can be attributed to the continuous increase in resistance to adsorption between the adsorbent and the adsorbate medium with the rise in the concentration of Cd^2^.

The escalating resistance to adsorption implies that Cd^2+^ can more effectively occupy adsorption sites on the surfaces of XIM and BIM until these sites become saturated. This saturation point marks the limit at which the adsorption capacity of the biochar materials reaches its maximum. The data presented in Figure 6a,b highlight the intricate interplay between initial Cd^2+^ concentration, adsorption capacity, and adsorption kinetics, underscoring the importance of understanding these factors in optimizing the efficiency of Cd^2+^ removal using biochar materials [41].

Table 4 reveals that the fitting of XIM to the Langmuir adsorption model yielded correlation coefficients of *R*^2^ = 0.9879, whereas the Freundlich model resulted in *R*^2^ = 0.9607. For BIM, the Langmuir adsorption model produced correlation coefficients of *R*^2^ = 0.9909, and the Freundlich model gave *R*^2^ = 0.9798. The correlation coefficients exceeding 0.95 indicate a strong fit for both the Langmuir and Freundlich models in describing the adsorption processes of Cd^2+^ by XIM and BIM. The high values of *R*^2^ suggest that both models effectively capture the behavior of the XIM and BIM processes with respect to Cd^2+^ adsorption, indicating a combination of surface chemisorption and multilayer adsorption mechanisms at play. The adsorption process of Cd^2+^ by ordinary distiller’s grains biochar is more in line with the Langmuir model, indicating that the adsorption process is single-layer adsorption [34]. The consistency of the Langmuir and Freundlich models with the experimental data underscores their utility in elucidating the adsorption characteristics of the biochar materials. It provides valuable insights into the adsorption mechanisms of removing Cd^2+^ from aqueous solutions.

### 3.7. Adsorption Kinetic Analysis

**Figure 7:**
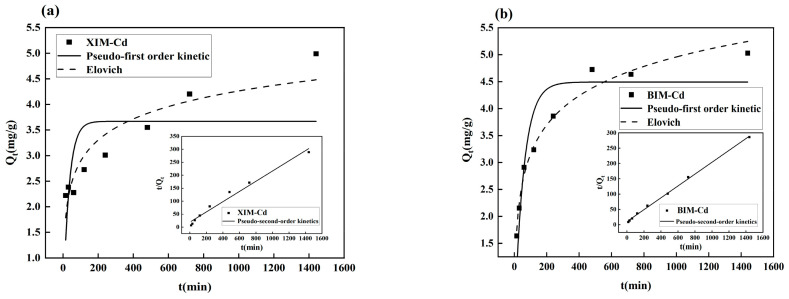
Kinetic adsorption fitting curves of adsorption of Cd^2+^ by XIM (**a**) and BIM (**b**).

Figure 7a displays the adsorption kinetic model fitting plot of XIM for Cd^2+^. The correlation coefficient of the pseudo-second-order kinetic model fitting for the adsorption kinetics of XIM with Cd^2+^ is calculated as 0.9797 (Table 5). In contrast, the lower correlation coefficients for the pseudo-first-order kinetic and Elovich model fitting indicate that the pseudo-second-order kinetic modeling aligns more closely with the adsorption process of XIM for Cd^2+^, as evidenced by the higher correlation coefficient. This suggests that the adsorption of Cd^2+^ by XIM is mainly through chemical adsorption, which is similar to the biochar of ordinary distillers’ grains [34]. Figure 7b presents the kinetic model fitting of the adsorption of Cd^2+^ by BIM. The kinetic fitting results reveal that the correlation coefficients for the pseudo-first-order kinetic, pseudo-second-order kinetic, and Elovich model fits for the adsorption kinetics of Cd^2+^ by BIM are 0.8499, 0.9983, and 0.9805, respectively (Table 5). Both the pseudo-second-order kinetic and Elovich models provide better fits to the adsorption process, as indicated by correlation coefficients exceeding 0.95, indicating that the adsorption of Cd^2+^ by biochar is a heterogeneous process constrained by diffusion, with chemical adsorption being its primary adsorption mechanism.

The findings from the kinetic model fittings underscore the importance of selecting appropriate kinetic models to accurately describe the adsorption dynamics of Cd^2+^ by biochar materials. The higher correlation coefficients for the pseudo-second-order kinetic model in the case of XIM and the pseudo-second-order kinetic and Elovich models in the case of BIM suggest that these models offer better representations of the adsorption processes and highlight the complexity of the adsorption mechanisms involved.

### 3.8. Post-Adsorption Characterization Results and Analysis

**Figure 8:**
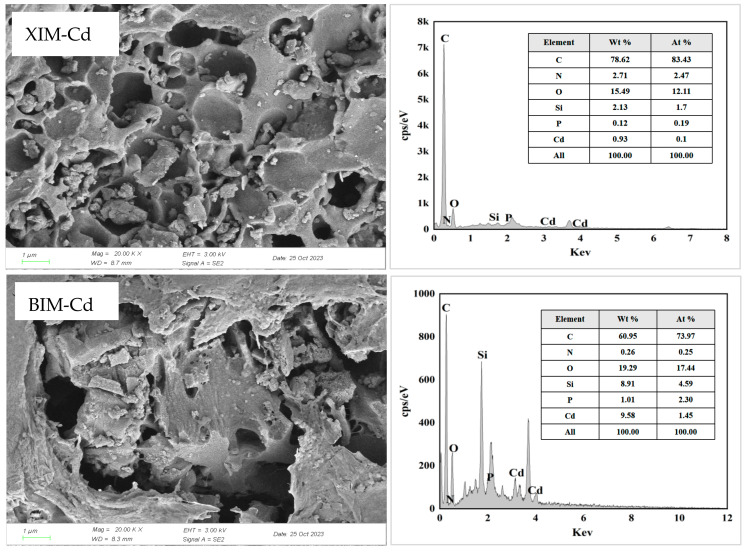
SEM + EDS analysis of XIM and BIM adsorption of Cd^2+^.

Figure 8 characterizes the morphological changes and elemental composition of XIM and BIM after the adsorption of Cd^2+^ using SEM–EDS analysis. The images reveal noticeable alterations in the pore structure of XIM and BIM following the adsorption of Cd^2+^. The surfaces of both materials show signs of collapse, deformation, and the presence of granular impurities, indicating a restructuring of the surface morphology due to the adsorption process. This result suggests that Cd ions have infiltrated the surface and inner walls of the pores of XIM and BIM [42]. The EDS spectroscopy analysis confirms the successful adsorption of Cd^2+^ on both the surface and within the structures of XIM and BIMq. The elemental analysis indicates that the content of elemental Cd in XIM is approximately 1.07%, whereas BIM accounts for around 11.15%. This discrepancy implies that BIM has a higher adsorption capacity for Cd^2+^ than XIM, as reflected in the higher content of elemental Cd detected in BIM. These findings highlight the effectiveness of both XIM and BIM in adsorbing Cd^2+^ from solution and generate insights into the distribution and concentration of Cd ions within the biochar materials.

**Figure 9:**
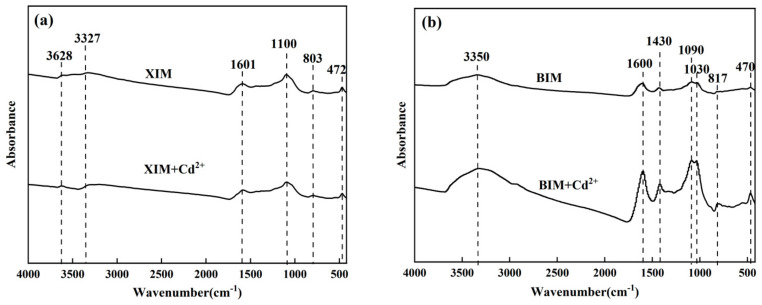
FTIR spectra of XIM (**a**) and BIM (**b**) before and after adsorption of Cd^2+^.

The changes in functional groups following the adsorption of Cd^2+^ onto XIM (XIM + Cd^2+^) were investigated using FTIR spectroscopy, and the findings are presented in Figure 9a. The analysis did not reveal any significant alterations in the peaks corresponding to functional groups following the adsorption of Cd^2+^ on XIM. This observation led to the hypothesis that the adsorption mechanism of XIM with Cd^2+^ may not involve ion exchange processes. In Figure 9b, the BIM + Cd^2+^ sample was analyzed using infrared spectroscopy to assess the changes in functional groups before and after Cd^2+^ adsorption on BIM. The results show enhancements in the peaks corresponding to various functional groups, including -OH (at around 3350 cm^−1^), carboxyl group C=O (at approximately 1600 cm^−1^), C=C (at about 1430 cm^−1^), -C-O (in the range of 1030–1090 cm^−1^), and Si-O-Si (at 817 and 470 cm^−1^) following the adsorption of Cd^2+^ on the BIM surface. The increased intensities of these peaks suggest that the functional groups on the surface of BIM, such as -OH, C=O, -C-O, among others, may have interacted with Cd ions, leading to changes in their vibrational modes. This phenomenon indicates a potential exchange of functional groups on the surface of BIM with Cd ions. The observed enhancements in the peaks associated with various functional groups after Cd^2+^ adsorption on BIM imply a surface modification process involving interactions between the functional groups on BIM and Cd species, possibly through chemical reactions that result in the exchange of functional groups with Cd ions [43].

**Figure 10:**
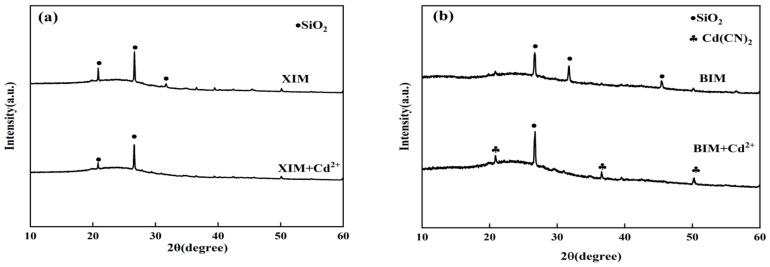
XRD spectra of XIM (**a**) and BIM (**b**) after adsorption of Cd^2+^.

Figure 10 presents the XRD patterns of XIM and BIM after the adsorption of Cd^2+^. In Figure 10a, the XRD pattern of XIM following Cd^2+^ adsorption did not exhibit significant changes compared to the pattern before adsorption. No new diffraction peaks were observed, suggesting that the structure of XIM remained relatively unchanged after interacting with Cd^2+^. In contrast, in Figure 10b, distinct diffraction peaks corresponding to Cd(CN)_2_ were detected at 2θ values of 20.88, 36.56, and 50.28 after the adsorption of Cd^2+^ by BIM. This observation suggests BIM underwent ion exchange processes with Cd^2+^ during adsorption. The appearance of characteristic peaks of Cd(CN)_2_ in the XRD pattern of BIM post-Cd^2+^ adsorption provides evidence of the interaction between BIM and Cd^2+^, leading to the formation of Cd(CN)_2_ compounds due to ion exchange mechanisms.

## 4. Discussion

Previous studies have demonstrated that the primary adsorption mechanisms of biochar-immobilized microorganisms include surface physical adsorption, electrostatic attraction, void retention, ion exchange, and surface precipitation [41,44]. The results obtained from batch adsorption experiments indicated that both XIM and BIM, in which *Lactobacillus plantarum* was immobilized on distiller’s grains, could adsorb Cd^2+^ ions. The fitting models applied for isothermal and kinetic adsorption analyses revealed distinct adsorption behaviors. Specifically, the adsorption of Cd^2+^ by XIM followed the Langmuir and Freundlich models, highlighting surface and porous adsorption processes [45]. In contrast, the adsorption of Cd^2+^ by BIM adhered to the Langmuir and Freundlich models while also displaying a good fit with the pseudo-second-order kinetic and Elovich models. These results suggest that the adsorption mechanisms of BIM on Cd^2+^ may involve a combination of surface adsorption and intra-particle diffusion processes [46].

The SEM-EDS, FTIR, and XRD analyses showed no obvious crystal precipitation was formed between XIM and Cd^2+^ in solution. The characteristic peaks of FTIR did not have obvious changes from those before adsorption, but the element Cd appeared in the EDS pattern. It was speculated that the adsorption mechanism of Cd^2+^ adsorption by XIM was mainly surface physical adsorption and pore interception, which agreed with the results of the isothermal adsorption model and kinetic adsorption model. The SEM analysis showed that the surface of BIM-adsorbed Cd^2+^ had obvious changes, and its reticulated pores were covered with granular materials. FTIR analysis revealed that the characteristic peaks of the functional groups on the surface of BIM-adsorbed Cd^2+^ were significantly enhanced, and it was speculated that BIM might have ion-exchanged with Cd^2+^. Meanwhile, the XRD results showed that Cd(CN)_2_ was generated after the adsorption of Cd^2+^ on BIM, and the EDS pattern showed that Cd element appeared after the adsorption, which indicated that Cd^2+^ might have reacted with the -CN in BIM to produce Cd(CN)_2_ crystals attached to the BIM surface.

The outcomes of SEM-EDS, FTIR, and XRD analyses revealed distinctive findings regarding the interactions between XIM and Cd^2+^ ions in solution. No significant crystal precipitation was observed between XIM and Cd^2+^, as evidenced by SEM and EDS results. Although FTIR spectral analysis showed no clear changes in characteristic peaks before and after Cd^2+^ adsorption, the presence of Cd in the EDS pattern indicated adsorption of the metal onto XIM. It was proposed that the primary mechanism of Cd^2+^ adsorption by XIM involved surface physical adsorption and pore interception, aligning with the observations from isothermal and kinetic adsorption models. Conversely, SEM images of BIM following Cd adsorption exhibited noticeable alterations, with reticulated pores covered by granular materials. FTIR analysis indicated significant enhancements in characteristic peaks of functional groups on the surface of BIM post-Cd^2+^ adsorption, suggesting a potential ion exchange interaction between BIM and Cd^2+^. XRD results displayed the formation of Cd(CN)_2_ following Cd^2+^ adsorption on BIM. At the same time, EDS patterns confirmed the presence of Cd, implying a potential reaction between Cd^2+^ and -CN groups in BIM, leading to the attachment of Cd(CN)_2_ crystals on the BIM surface.

Therefore, the comprehensive analysis of the adsorption mechanism of cadmium by XIM and BIM revealed that a combination of factors affected the process. The mechanism of cadmium adsorption involves physical diffusion adsorption, ion exchange, and surface precipitation.

## 5. Conclusions

(1) With increasing dosages of XIM and BIM, the unit adsorption amount of Cd^2+^ gradually decreases, whereas the adsorption efficiency shows an upward trend. The adsorption of Cd^2+^ increases with higher solution pH, reaching maximum levels of adsorption amount and efficiency at a pH of 6.0.
(2) The adsorption of Cd by XIM and BIM exhibited an increase with the concentration of Cd^2+^. XIM and BIM reached equilibrium at a Cd^2+^ concentration of 60 mg/L. BIM consistently demonstrated significantly higher Cd^2+^ adsorption than XIM across high Cd^2+^ concentrations.
(3) The adsorption of Cd^2+^ by both XIM and BIM increased gradually with the extension of time. Cd^2+^ adsorption by XIM primarily involved surface and porous adsorption mechanisms. In contrast, the adsorption process of Cd^2+^ by BIM encompassed surface adsorption and intra-particle diffusion.
(4) The adsorption of Cd^2+^ by XIM predominantly entailed physical adsorption and void retention. Conversely, the adsorption mechanism of Cd^2+^ by BIM predominantly involved the formation of Cd(CN)_2_ with Cd^2+^. Furthermore, the specific surface area, pore size, and functional group content inherent to XIM and BIM materials collectively affected their Cd^2+^ d adsorption processes.

## Figures and Tables

**Table 1:** Experimental conditions of Cd^2+^ adsorption.

Absorbent Material	Experimental Projects	Variable Settings	Experimental Condition
XIM BIM	One-way experiment	Dosage: 10, 20, 40, 60, 80, 100, 120 mg	Cd^2+^ Concentration: 20 mg/L, pH = 6.0, Reaction time: 1440 min (24 h)
		pH: 3, 4, 5, 6, 7	Cd^2+^ Concentration: 20 mg/L, XIM/BIM dosage: 50 mg, Reaction time: 1440 min (24 h)
	Kinetic experiment	Reaction time: 15, 30, 60, 120, 240, 480, 720, 1440	Cd^2+^ Concentration: 20 mg/L, pH = 6.0, XIM/BIM dosage: 50 mg
	Isothermal adsorption experiment	Cd^2+^ solution: 5, 10, 20, 40, 60, 80 mg/L	pH = 6.0, XIM/BIM dosage: 50 mg, Reaction time: 1440 min (24 h)

**Table 2:** Physicochemical properties of biochars.

Biochar	pH	Specific Area (m^2^/g)	Total Pore Volume (cm^3^/g)	Average Pore Diameter (nm)
BC	9.94	5.3207	0.0210	15.8109
XIM	7.23	5.8500	0.0195	13.3140
BIM	7.78	7.0270	0.0146	8.3068

**Table 3:** Adsorption amount and adsorption efficiency of Cd^2+^ under various adsorption experimental conditions.

Experimental Projects	Variable	Variable Settings	Adsorption Amount (mg/g)		Adsorption Efficiency (%)	
			XIM	BIM	XIM	BIM
One-way experiment	Dosage	10 mg	11.42 ± 0.93	14.09 ± 1.01	33.18% ± 1.93%	42.17% ± 2.66%
		20 mg	7.80 ± 0.28	9.25 ± 0.11	44.60% ± 1.18%	53.24% ± 0.52%
		40 mg	5.34 ± 0.13	5.47 ± 0.20	60.84% ± 1.28%	62.50% ± 2.15%
		60 mg	4.67 ± 0.30	3.87 ± 0.13	79.61% ± 5.23%	66.31% ± 2.17%
		80 mg	3.86 ± 0.11	3.12 ± 0.06	87.51% ± 2.30%	70.85% ± 1.19%
		100 mg	3.29 ± 0.03	2.61 ± 0.08	93.44% ± 1.02%	74.20% ± 2.14%
		120 mg	2.82 ± 0.02	2.20 ± 0.02	95.91% ± 0.64%	74.91% ± 0.59%
	pH	3	1.85 ± 0.26	4.09 ± 0.17	23.42% ± 3.30%	51.72% ± 2.03%
		4	4.42 ± 0.05	4.90 ± 0.08	58.38% ± 0.72%	64.56% ± 0.86%
		5	4.71 ± 0.17	4.97 ± 0.13	23.42% ± 1.96%	51.72% ± 1.39%
		6	4.73 ± 0.10	4.93 ± 0.18	58.38% ± 1.45%	64.56% ± 2.12%
		7	4.53 ± 0.05	4.80 ± 0.16	63.01% ± 0.50%	66.91% ± 1.83%
Kinetic experiment	Time	15 min	2.22 ± 0.09	1.64 ± 0.07	29.44% ± 1.30%	21.78% ± 0.86%
		30 min	2.38 ± 0.05	2.15 ± 0.28	31.51% ± 0.75%	28.57% ± 3.63%
		60 min	2.28 ± 0.09	2.91 ± 0.18	30.26% ± 1.25%	38.76% ± 2.25%
		120 min	2.73 ± 0.07	3.23 ± 0.17	36.21% ± 0.98%	42.91% ± 2.12%
		240 min	3.01 ± 0.11	3.86 ± 0.22	39.91% ± 1.49%	51.25% ± 2.68%
		480 min	3.55 ± 0.49	4.73 ± 0.62	47.06% ± 6.43%	62.71% ± 8.20%
		720 min	4.20 ± 0.63	4.64 ± 0.31	55.55% ± 8.30%	61.69% ± 4.14%
		1440 min	4.99 ± 0.10	5.03 ± 0.05	66.10% ± 1.40%	66.93% ± 0.67%
Isothermal adsorption experiment	Cd^2+^ solution	5 mg/L	1.52 ± 0.01	1.41 ± 0.03	80.52% ± 0.80%	74.98% ± 1.51%
		10 mg/L	3.15 ± 0.14	2.78 ± 0.05	81.47% ± 3.50%	72.55% ± 1.15%
		20 mg/L	5.03 ± 0.11	5.08 ± 0.04	66.27% ± 1.39%	67.09% ± 0.66%
		40 mg/L	6.51 ± 0.14	8.66 ± 0.44	44.51% ± 0.96%	59.25% ± 3.13%
		60 mg/L	8.40 ± 0.30	12.23 ± 0.10	40.03% ± 1.42%	58.42% ± 0.55%
		80 mg/L	8.41 ± 0.34	12.82 ± 1.33	33.03% ± 1.34%	50.79% ± 5.33%

**Table 4:** Fitting parameters of isothermal adsorption models of adsorption of Cd^2+^.

Absorbent Material	Langmuir			Freundlich		
	*q_m_*	*K_L_*	*R* ^2^	*K*_F_	1/*n*	*R* ^2^
XIM	11.64	0.0357	0.9879	1.0136	0.4991	0.9607
BIM	27.43	0.0118	0.9909	0.6075	0.7110	0.9798

**Table 5:** Kinetic model fitting parameters of adsorption of Cd^2+^ by XIM and BIM.

Absorbent Material	Pseudo-First Order Kinetic			Pseudo-Second Order Kinetics			Elovich		
	*K*_1_	*q_e_*	*R* ^2^	*K*_2_	*q_e_*	*R* ^2^	*α*	*β*	*R* ^2^
XIM	0.0305	3.67	0.3434	0.0020	5.08	0.9797	0.7484	1.6735	0.8701
BIM	0.0175	4.49	0.8499	0.0035	5.17	0.9983	0.4224	1.2674	0.9805

## Data Availability

All data is presented in this study and thus contained within the article. There are no other available data in any publicly accessible repository.
